# Separated right and left ventricular excitation during right ventricular septal pacing in a patient with narrow QRS wave: a case report

**DOI:** 10.1186/1752-1947-8-158

**Published:** 2014-05-21

**Authors:** Takanori Yaegashi, Hiroshi Furusho, Akio Chikata, Soichiro Usui, Shuichi Kaneko, Masakazu Yamagishi, Masayuki Takamura

**Affiliations:** 1 Department of Cardiology, Kanazawa University Hospital, 13-1, Takara-machi, Kanazawa 920-8641, Japan

## Abstract

**Introduction:**

Right ventricular septal pacing is thought to be better than right ventricular apical pacing for shortening the QRS duration and for preserving left ventricular function. However, right ventricular septal pacing may not be effective in all cases. In this case report, we present a rare case in which right ventricular septal pacing induced thoroughly separated right and left ventricular excitation despite the presence of a relatively narrow QRS wave during atrium-only pacing.

**Case presentation:**

We report a case of 63-year-old Japanese man with cardiomyopathy with an implantable cardioverter defibrillator placement for ventricular tachycardia. Three years after implantation, he developed second-degree atrio-ventricular block. Therefore, atrio-ventricular sequential pacing was started; then his heart failure was much worsened. His electrocardiogram showed a dissociated biphasic QRS wave during right ventricular high-septal pacing, despite the presence of a non-fragmented QRS morphology during atrium-only pacing. An activation map during right ventricular high-septal pacing showed that right ventricular conduction started at the pacing site and ended at the right ventricular basal inferior site. Subsequently after a 10ms interval, left ventricular conduction started at the left ventricular posteroseptum and ended at the left ventricular lateral wall. These data indicate that during right ventricular high-septal pacing, the first component of the QRS wave supposedly reflects only right ventricular excitation and the second component only left ventricular excitation. Also due to the intracardiac electrograms, it was assumed that this phenomenon was caused by transversely limited severe transseptal conduction disturbance.

**Conclusion:**

It should be noted that even ventricular septal pacing could evoke harmful interventricular dyssynchrony due to transversely limited severe septal conduction disturbance, despite the presence of a relatively narrow QRS wave.

## Introduction

A prolonged duration of the QRS complex on electrocardiogram is associated with adverse prognosis not only in patients with cardiac diseases [1,2] but also in the general population [3]. Right ventricular (RV) septal pacing is thought to be better than RV apical pacing for shortening the QRS duration and for preserving left ventricular (LV) function [4,5]. However, there are no apparent data suggesting that RV septal pacing is better than RV apical pacing for patients’ prognosis.

In this report, we present a case in which RV septal pacing induced thoroughly separated RV and LV excitation and contraction.

## Case presentation

**Figure 1:**
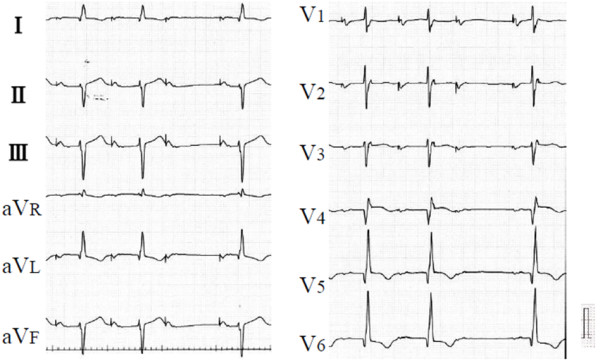
**Twelve-lead electrocardiogram during atrium-only pacing.** The patient experienced a Wenckebach-type second-degree atrioventricular block during atrium-only pacing at 60bpm (beats per minute). The width of the QRS wave was 120ms.

**Figure 2:**
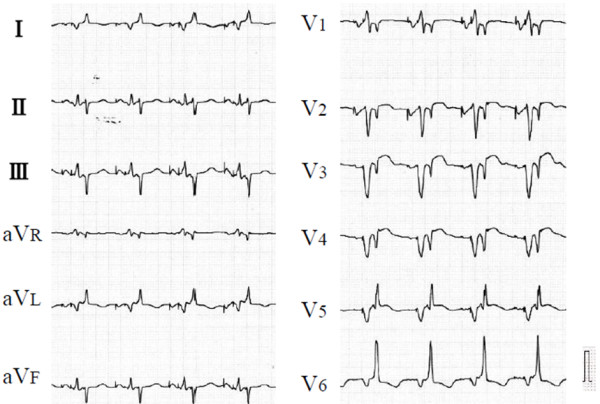
**Twelve-lead electrocardiogram during atrioventricular sequential pacing.** During atrioventricular sequential pacing at 70bpm (beats per minute) with right ventricular high-septal pacing, the QRS wave showed dissociated biphasic morphology. The width of the first component of the QRS wave was 110ms, and that of the second component was 102ms.

A 63-year-old Japanese man who had cardiomyopathy of unknown etiology experienced ventricular tachycardia, and an implantable cardioverter defibrillator (ICD) was thus implanted. The ventricular lead was fixed on his RV high septum, because a low-voltage area extended across his mid- to low-ventricular septum. The pacing mode of the ICD was programmed to AAI mode. The width of his own QRS wave was 120ms. His echocardiogram showed a severely dilated and diffuse hypokinetic left ventricle. His septal wall was thin and high-echoic. Cardiac sarcoidosis was suspected, but the diagnostic criteria could not be fulfilled. Three years after ICD implantation, he developed dyspnea until he experienced New York Heart Association (NYHA) class III heart failure. A Wenckebach-type second-degree atrioventricular block was observed during atrium-only pacing at 60 beats per minute (Figure 1), therefore, the pacing mode was programmed to DDDR mode. After changing the pacing mode, his symptoms apparently worsened, and he was admitted the following week. He experienced NYHA class IV heart failure, and his electrocardiogram showed dissociated biphasic QRS morphology (Figure 2). The width of the first component of the QRS wave was 110ms, and that of the second component was 102ms. His echocardiogram showed severe interventricular dyssynchrony. LV ejection occurred 150ms later than did RV ejection.

**Figure 3:**
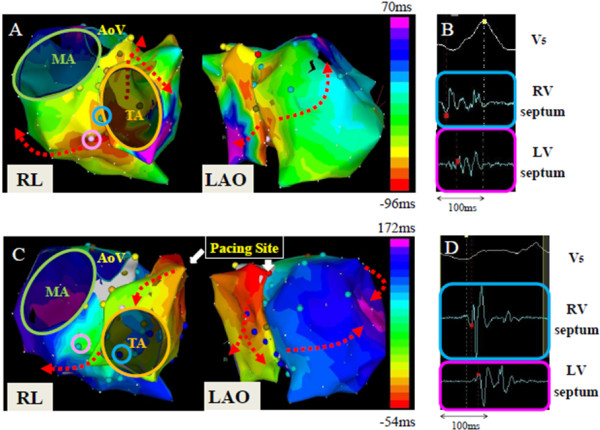
**Isochronal endocardial activation map generated by the CARTO™ system. Panel A**: Biventricular mapping of his own QRS wave showed that ventricular conduction started at the basal septum, and both left ventricular and right ventricular excitation ended simultaneously within 166ms. **Panel B**: The local electrograms at the light-blue tag on the right ventricular septum (light-blue circle, ○, in Panel A) and at the pink tag on the left ventricular septum (pink circle, ○, in Panel A) showed fragmentation. **Panel C**: Biventricular mapping during right ventricular high-septal pacing showed that ventricular conduction started at the pacing site (white arrow in Panel C), and after the completion of the whole right ventricular excitation, left ventricular conduction started from the septum and ended at the left ventricular lateral wall within 226ms after the pacing. **Panel D**: The local electrograms at the light-blue tag on the right ventricular septum (light-blue circle, ○, in Panel C) and at the pink tag on the left ventricular septum (pink circle, ○, in Panel C) showed double potentials that supposedly reflect separated right ventricular and left ventricular excitations. Abbreviations: AoV, aortic valve; LAO, right anterior oblique projection; LV, left ventricular; MA, mitral annulus; RL, right lateral projection; RV, right ventricular; TA, tricuspid annulus.

**Figure 4:**
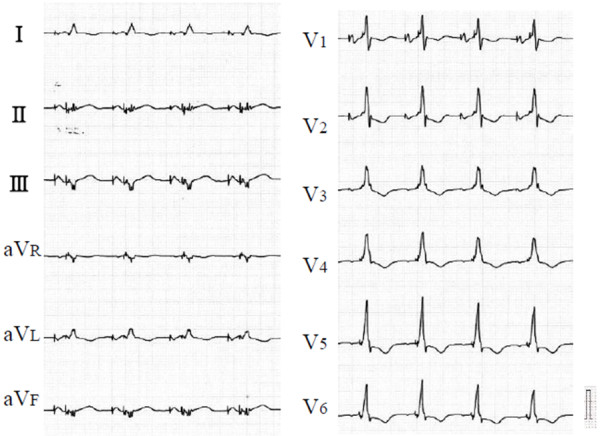
**Twelve-lead electrocardiogram after cardiac resynchronization therapy.** The width of the QRS wave was 172ms.

Endocardial substrate mapping by the CARTO™ XP system (Biosense Webster Inc., Diamond Bar, CA, USA) showed a low-voltage area extended across his basal to mid-ventricular septum. Activation mapping of his own QRS wave (Figure 3A) showed that ventricular conduction started at his mid-septum, and both LV and RV excitation ended simultaneously within 166ms. However, activation mapping during RV high-septal pacing (Figure 3C) showed that RV conduction started at the pacing site and ended at the RV basal inferior site within 83ms after the pacing stimulus. Subsequently after a 10ms interval, LV conduction started at his basal posterior septum and ended at the lateral wall within 226ms after the pacing stimulus. These data show that in RV high-septal pacing, the first component of the QRS wave supposedly reflects only RV excitation originating at the RV high septum, and the second component reflects only LV excitation originating at the LV posteroseptum. His electrocardiogram (ECG) also showed biphasic QRS morphology during LV lateral or RV apical pacing. This interventricular dyssynchrony was improved by biventricular pacing, therefore, his ICD was upgraded to cardiac resynchronization therapy with defibrillator (CRTD; Figure 4). Two years after upgrading to CRTD, he showed good hemodynamic conditions and improved to NYHA class II.

## Discussion

It was reported that RV septal pacing might induce intraventricular LV dyssynchrony causing severe LV ejection fraction deterioration and symptoms of congestive heart failure [6]. However, septal pacing-induced interventricular dyssynchrony has not been reported. In this case, an ECG of the patient during RV high-septal pacing as well as during LV lateral or RV apical pacing showed a biphasic QRS complex, and this QRS morphology supposedly reflects completely separated RV and LV excitations, although the intrinsic QRS morphology in this case showed only a mild intraventricular conduction disturbance pattern.

In a previous study on patients with left bundle-branch block [7], two patterns of initiation of LV septal activation were observed: (1) via slow conduction through the left bundle branch and (2) via right-to-left transseptal activation. In the former pattern, the earliest LV activation occurred in the mid-septum by slow conduction through the left posterior fascicle, whereas in the latter pattern, the earliest LV activation occurred in the high septum. In the present case, the earliest LV activation started at the mid-septum during atrium-only pacing and at the basal posterior septum during the RV high-septal pacing. In addition, while intracardiac mapping was performed, fragmented potentials were recorded in the posteroseptal area during atrium-only pacing (Figure 3B), and double potentials were recorded in the same area during RV septal pacing (Figure 3D); this shows that severe conduction disturbance may have been present around this region on the working myocardium, and that myocardial excitation rose from the RV septal pacing site conducted very slowly through this damaged area transversely.

During atrium-only pacing, RV and LV excitation started and ended almost simultaneously within 166ms. During RV septal pacing, RV and LV excitation occurred sequentially; however, intraventricular conduction durations of the right and left ventricles measured by activation mapping were comparatively shorter than those during atrium-only pacing (83ms and 133ms in the right and left ventricles, respectively); therefore, it seems that each intraventricular conduction uses some intact cardiac conduction system.

## Conclusions

We experienced a rare case of idiopathic cardiomyopathy that showed a dissociated biphasic wide QRS complex and completely separated RV and LV contractions during RV high-septal pacing due to transversely limited severe transseptal conduction disturbance, despite the presence of a relatively narrow QRS wave during atrium-only pacing. Although RV septal pacing can usually achieve more physiological ventricular contraction than RV apical pacing, it should be noted that even septal pacing could evoke harmful interventricular dyssynchrony.

## Consent

Written informed consent was obtained from the patient for publication of this case report and any accompanying images. A copy of the written consent is available for review by Editor-in-Chief of this journal.

## Competing interests

The authors declare that they have no competing interests.

## Authors’ contributions

TY analyzed and interpreted patient data including cardiac electrophysiological mapping, and was the major contributor in writing the manuscript. HF and AC performed most medical patient care including ICD implantation and management. SU and MT contributed in electrophysiological and circulatory interpretation. SK and MY provided major diagnostic and therapeutic suggestions, especially on patient care. All authors read and approved the final manuscript.
